# Assessing the Racial and Socioeconomic Disparities in Postpartum Depression Using Population-Level Hospital Discharge Data: Longitudinal Retrospective Study

**DOI:** 10.2196/38879

**Published:** 2022-10-17

**Authors:** Star Liu, Xiyu Ding, Anas Belouali, Haibin Bai, Kanimozhi Raja, Hadi Kharrazi

**Affiliations:** 1 Johns Hopkins University School of Medicine Baltimore, MD United States; 2 Johns Hopkins University Bloomberg School of Public Health Baltimore, MD United States

**Keywords:** health disparity, hospital discharge summary, phenotyping, data quality, vulnerable population, postpartum depression, maternal health

## Abstract

**Background:**

In the United States, >3.6 million deliveries occur annually. Among them, up to 20% (approximately 700,000) of women experience postpartum depression (PPD) according to the Centers for Disease Control and Prevention. Absence of accurate reporting and diagnosis has made phenotyping of patients with PPD difficult. Existing literature has shown that factors such as race, socioeconomic status, and history of substance abuse are associated with the differential risks of PPD. However, limited research has considered differential temporal associations with the outcome.

**Objective:**

This study aimed to estimate the disparities in the risk of PPD and time to diagnosis for patients of different racial and socioeconomic backgrounds.

**Methods:**

This is a longitudinal retrospective study using the statewide hospital discharge data from Maryland. We identified 160,066 individuals who had a hospital delivery from 2017 to 2019. We applied logistic regression and Cox regression to study the risk of PPD across racial and socioeconomic strata. Multinomial regression was used to estimate the risk of PPD at different postpartum stages.

**Results:**

The cumulative incidence of PPD diagnosis was highest for White patients (8779/65,028, 13.5%) and lowest for Asian and Pacific Islander patients (248/10,760, 2.3%). Compared with White patients, PPD diagnosis was less likely to occur for Black patients (odds ratio [OR] 0.31, 95% CI 0.30-0.33), Asian or Pacific Islander patients (OR 0.17, 95% CI 0.15-0.19), and Hispanic patients (OR 0.21, 95% CI 0.19-0.22). Similar findings were observed from the Cox regression analysis. Multinomial regression showed that compared with White patients, Black patients (relative risk 2.12, 95% CI 1.73-2.60) and Asian and Pacific Islander patients (relative risk 2.48, 95% CI 1.46-4.21) were more likely to be diagnosed with PPD after 8 weeks of delivery.

**Conclusions:**

Compared with White patients, PPD diagnosis is less likely to occur in individuals of other races. We found disparate timing in PPD diagnosis across different racial groups and socioeconomic backgrounds. Our findings serve to enhance intervention strategies and policies for phenotyping patients at the highest risk of PPD and to highlight needs in data quality to support future work on racial disparities in PPD.

## Introduction

### Background

In the United States, >3.6 million deliveries occur each year. Among them, up to 20% (approximately 700,000) of women experience postpartum depression (PPD) according to the Centers for Disease Control and Prevention [1,2]. However, this rate could be underestimated because of low screening rates, high proportions of unreported or undiagnosed cases, lack of help-seeking behavior, and cultural stigma [3-7]. Thus, it is challenging to phenotype patients with the highest risk for PPD. PPD can occur anytime in the following year after delivery. The earlier the diagnosis, the more favorable the outcomes of the treatment. PPD can negatively affect women’s postpartum health and child development if left untreated [8]. Given its detrimental impacts, we must address how disparate PPD outcomes and complications could be attributed to demographic, socioeconomic, and behavioral factors [9,10].

Existing literature has shown that factors such as race, socioeconomic status, and history of substance abuse are associated with the differential risks of PPD. One such study found that the odds of hospital-based PPD (emergency room and inpatient visits, as defined in the study) were highest among the Black population and lowest among the Asian population [11]. Similarly, other researchers have found that African American and Latina mothers from small towns, cities, and rural areas are more vulnerable to PPD compared with White mothers [12]. Accrued evidence suggests that compared with White women, women of other races, women of lower socioeconomic status, those not living in urban areas, and those with a history of depression are more likely to be diagnosed with PPD [13]. However, limited research has considered how certain factors could have temporal associations with the outcome [14].

The disparity in PPD is also attributed to sociocultural factors. Previous research has documented that racial and ethnic groups perceive PPD differently [15-18]. Although consensus on which racial group exhibits greater help-seeking behavior is lacking, hesitancy to seek treatment is the common theme. Across White, Hispanic, Asian, and women of other races, many do not believe that they warranted treatment for PPD [3,4]. Social and cultural stigmas may have contributed to this perception to varying degrees. Those who seek help would be diagnosed and treated early on, and those who are more reluctant are more likely to develop adverse outcomes. The entirety of cultural perception of PPD is difficult to assess; however, its impact cannot be overlooked in understanding the racial disparity in PPD and its timing.

### Objective

PPD screening and intervention strategies should not come as a one-size-fits-all approach, but rather be built upon the knowledge of disparate risks and timing of PPD. To address this gap, we investigated the racial and ethnic disparities in the risk and timing of PPD diagnosis using longitudinal statewide hospital discharge data.

## Methods

### Data Sources and Study Population

**Figure 1:**
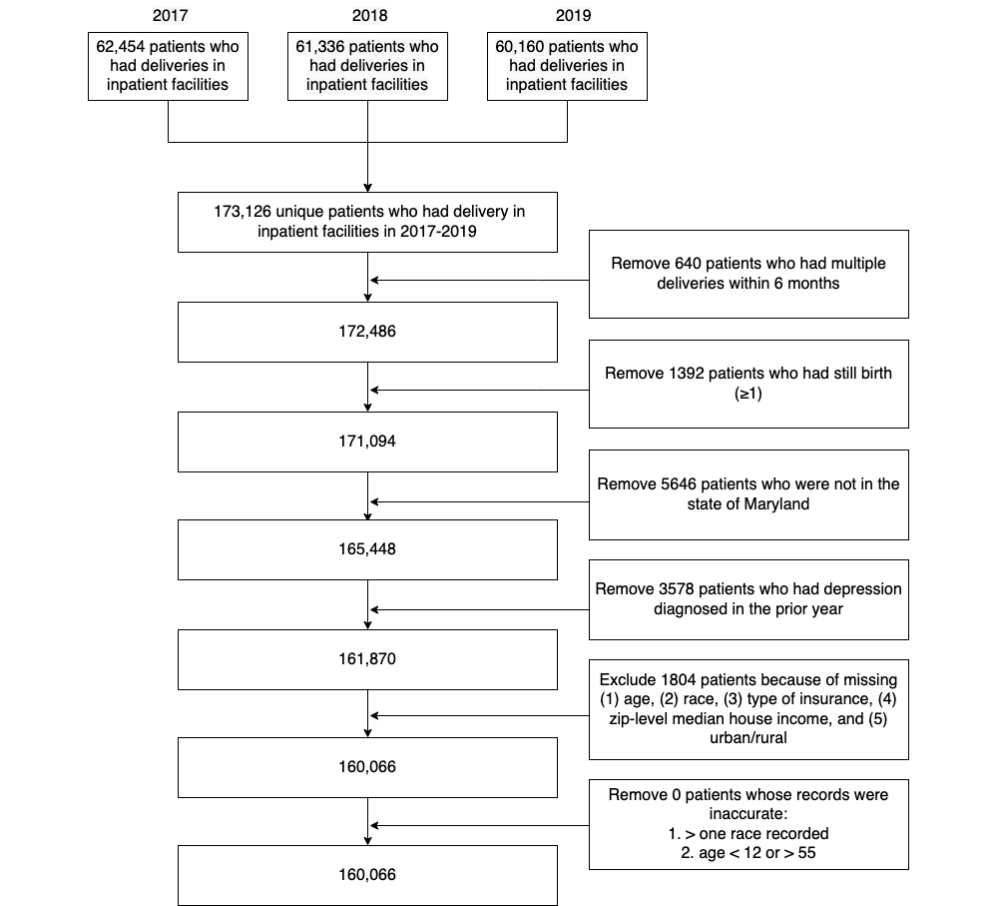
CONSORT (Consolidated Standards of Reporting Trials) diagram depicting the study population selection process.

The Healthcare Cost and Utilization Project (HCUP) data contain the largest longitudinal collection of all-payer, encounter-level data in the United States [19]. In this study, we used the HCUP Maryland state data sets (ie, hospital-based care including hospital inpatient, emergency department, and ambulatory care services) from 2016 to 2019. We only included individuals whose sex was registered as female. A total of 173,126 females had a hospitalization for delivery and had at least one inpatient postpartum visit within the outcome time frame (2017 to 2019). According to the World Health Organization, extremely preterm births are births that occurred before 28 weeks (approximately 7 months) of pregnancy [20]. We excluded those who had multiple deliveries within 6 months because they were more likely to be attributed to data inaccuracies than preterm births. As we were only interested in PPD among females with live births, 1392 females with pregnancy terminations (including stillbirths) were excluded. We then excluded 5646 individuals who had hospital encounters outside of Maryland. As studies have shown that a history of depression increases the likelihood of PPD, we excluded those who had a depression diagnosis a year before their delivery encounter. For example, females who delivered in 2017 were filtered for a depression diagnosis in 2016. Finally, 1804 patients were excluded owing to missingness of data on age, race, insurance type, zip-level median household income, or urbanicity. The final study population included 160,066 patients (Figure 1).

### Dependent Variables

The outcomes of interest were PPD diagnosis (hereon referred to as PPD) and the time to diagnosis from childbirth. PPD was defined and identified based on the presence of selected International Classification of Diseases (ICD-10) diagnosis codes (Multimedia Appendix 1) for depression in the first 12 months after delivery [14]. The timing of PPD diagnosis was measured by the number of days after delivery.

### Independent Variables

The independent variables included age, race and ethnicity, marital status, zip-level median household income, primary insurance type (referred to as insurance), and residential area type (ie, urban vs rural). Race and ethnicity were categorized into 6 major groups: non-Hispanic White, non-Hispanic Black, non-Hispanic Asian or Pacific Islander, non-Hispanic Native American, Hispanic, and other (races). In this study, we refer to non-Hispanic White as White, non-Hispanic Black as Black, non-Hispanic Asian or Pacific Islander as Asian or Pacific Islander, and non-Hispanic Native American as Native American.

### Statistical Analysis

We fitted a multivariate logistic regression model to explore the association between PPD diagnosis and the covariates, which included age, race and ethnicity, marital status, zip-level median household income, primary insurance type, and the residential area type. We also included the Charlson Comorbidity Index score because existing literature points to the positive association between chronic conditions and postpartum mental illness [21]. We calculated the cumulative incidence of PPD over the 12-month postpartum period stratified by race and ethnicity. We then applied the Cox regression model, treating the outcome as a time-to-event variable, to examine the association between PPD and race and ethnicity over the postpartum period adjusted by the other covariates. We treated patients with no depression records by the end of the year as censored observations, and the time to censoring was calculated based on the date of the patient’s last encounter. We further performed a log-rank test to examine differences in the timing of PPD diagnosis for each race and ethnicity.

Guided by previous studies, we adopted the cutoffs for the timing of PPD diagnosis as within 4 weeks, 4 to 8 weeks, and beyond 8 weeks after delivery [22]. Using these temporal cutoffs, we conducted multinomial logistic regression to further explore the risk differences for patients of different ages, race and ethnicity, marital status, primary insurance type, and residential area type.

For all regression models, associated CIs and *P* values were calculated. *P* values of <.001 were deemed statistically significant.

### Sensitivity Analysis

We performed a sensitivity analysis to assess whether our findings were robust against the exclusion of having a history of depression (Multimedia Appendix 2). Additional logistic regression, Cox regression, and multinomial regression were performed by including patients who had prior depression (encounter records of any diagnosis of ICD-10 Clinical Modification codes of depression).

In the primary analysis, we defined the diagnosis of PPD based on the existence of any depression-related ICD-10 codes within 1 year after giving birth. However, HCUP data are at the hospital discharge summary level; thus, each observation contains all the information on one entire hospital stay, excluding the more granular data. Furthermore, there is no specific ICD-10 code for “baby blues” (referred to as short-lasting moodiness and sadness in mothers), which occurs in 80% of the women 2-3 days after childbirth. Therefore, based on the current data source, we did not distinguish women who had PPD from those who had “baby blues.” We performed additional logistic regression by excluding patients who were diagnosed with depression during the same hospital stay as the delivery.

All visualization and statistical analyses were conducted using R (version 4.1.2; R Foundation for Statistical Computing) and the package survival [23,24].

### Ethics Approval

The Johns Hopkins institutional review board determined this study as a nonhuman subject research. Under section 8 of the Health Insurance Portability and Accountability Act of the HCUP Data Use Agreement, it states that HCUP, which conforms to the definition of a limited data set, does not require institutional review board review.

## Results

### Diagnosis of PPD

Our study included 160,066 women who underwent a delivery hospitalization in Maryland from January 1, 2017, to December 31, 2019. Table 1 presents the demographic information of the study population grouped by the presence of PPD diagnosis. Of the study population, 40.63% (65,028/160,066) were White, 30.58% (48,953/160,066) were Black, 17.78% (28,465/160,066) were Hispanic, 6.72% (10,760/160,066) were Asian or Pacific Islander, 0.37% (590/160,066) were Native American, and 3.92% (6270/160,066) were of other races and ethnicities. We also stratified the population characteristics by race groups (Table S1 in Multimedia Appendix 2). Compared with White women, Black and Hispanic women had higher proportions of public insurance (54.5% and 64.8%, respectively) enrollees. Among all racial groups, the Black population had the highest proportion of individuals living in areas with <US $59,000 median household income (12,658/48,953, 25.86%).

**Figure 2:**
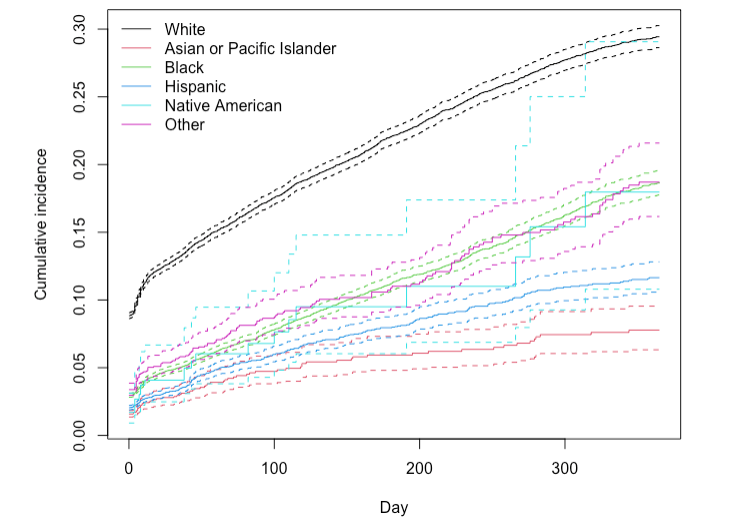
Cumulative incidence by race. Solid lines represent the cumulative incidence. Dashed lines represent the lower and upper bounds of the 95% CI.

**Figure 3:**
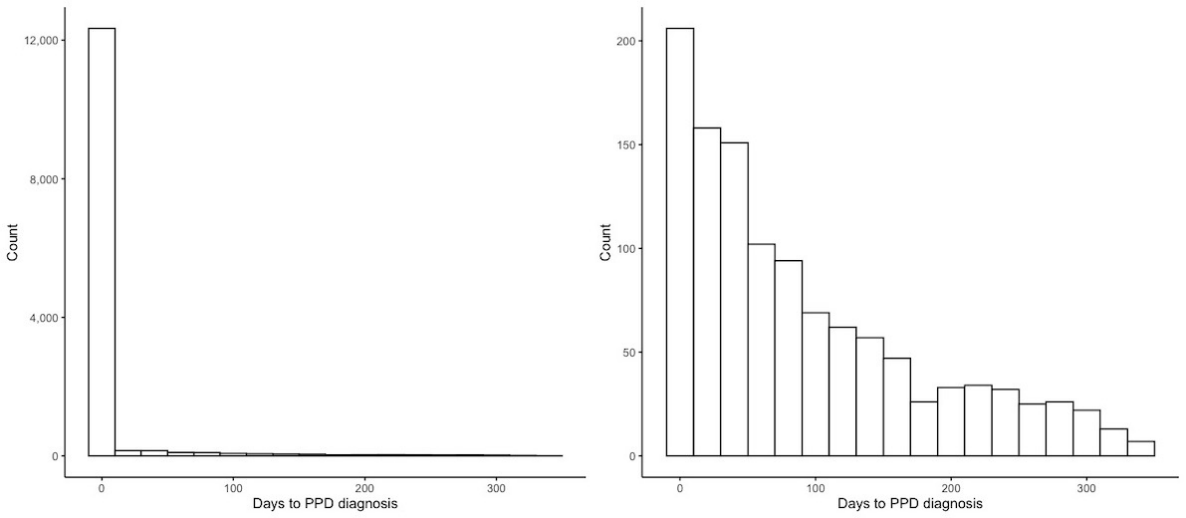
Time to postpartum depression (PPD) diagnosis (Left: including PPDs that occurred on the day of delivery; Right: excluding PPDs that occurred on the day of delivery).

The cumulative incidence of PPD in hospital-based care for women with no prior depression in the first year after delivery was 8.31% (13,297/160,066). Figure 2 compares the cumulative incidence of PPD among patients of different race and ethnicity. The cumulative incidence was the highest for White women (8779/65,028, 13.5%) and lowest for Asian and Pacific Islander women (248/10,760, 2.3%). Figure 3 shows the distribution of the timing of PPD diagnosis. Among those diagnosed, 91.1% (12,113/13,297) were diagnosed during the same hospital stay as childbirth (0 days to PPD diagnosis). The longest time to diagnosis after delivery was 349 days. Excluding patients who were diagnosed during the same hospital stay as childbirth, the median time to diagnosis was 68 days. The median time to diagnosis was 65 days for White women, 76 days for Black women, 51 days for Hispanic women, 66 days for Asian or Pacific Islander women, 20 days for Native American women, and 60 days for women of other races and ethnicities.

**Table 1:** Population demographics stratified by the presence of postpartum depression (PPD) diagnosis.

Variable		No PPD (n=146,769)		PPD (n=13,297)
**Age (years)**				
	Mean (SD)	30.0 (5.74)	29.9 (5.67)	
	Median (Range)	30.0 (12.0-55.0)	30.0 (13.0-51.0)	
**Race, n (%)**				
	White	56,249 (38.32)	8779 (66.02)	
	Black	46,059 (31.38)	2894 (21.76)	
	Hispanic	27,491 (18.73)	974 (7.32)	
	Asian or Pacific Islander	10,512 (7.16)	248 (1.86)	
	Native American	560 (0.38)	30 (0.22)	
	Other	5898 (4.02)	372 (2.8)	
**Marital status, n (%)**				
	Single	58,807 (40.07)	5635 (42.38)	
	Married	82,996 (56.55)	7100 (53.39)	
	Legally separated	755 (0.51)	114 (0.86)	
	Divorced	1156 (0.79)	235 (1.77)	
	Widowed	97 (0.07)	25 (0.19)	
	Other	2958 (2.01)	188 (1.41)	
**Zip-level median household income (US $), n (%)**				
	1-45,999	10,690 (7.28)	1176 (8.84)	
	46,000-58,999	12,906 (8.79)	1588 (11.94)	
	59,000-78,999	50,205 (34.2)	4028 (30.29)	
	>79,000	72,968 (49.72)	6505 (48.92)	
**Insurance type, n (%)**				
	Medicaid	59,279 (40.39)	4992 (37.54)	
	Medicare	311 (0.21)	113 (0.85)	
	Private insurance	77,556 (52.84)	7603 (57.18)	
	Self-pay	3136 (2.14)	125 (0.94)	
	No charge	2152 (1.47)	50 (0.38)	
	Other	4335 (2.95)	414 (3.11)	
**Urban or rural, n (%)**				
	Metropolitan areas of ≥1 million population	132,081 (89.99)	11,376 (85.55)	
	Metropolitan areas of 250,000 to 1 million population	7031 (4.79)	1172 (8.81)	
	Metropolitan areas of <250,000 population	4479 (3.05)	407 (3.06)	
	Urban population of 2500 to 19,999, adjacent to a metropolitan area	3176 (2.16)	342 (2.57)	
	Urban population of 2500 to 19,999, not adjacent to a metropolitan area	1 (0)	0 (0)	
	Completely rural or <2500 urban population, adjacent to a metropolitan area	1 (0)	0 (0)	
**Charlson Comorbidity Index score**				
	Mean (SD)	0.134 (0.430)	0.257 (0.586)	
	Median (Range)	0 (0-12.0)	0 (0-9.00)	

Table 2 shows the results of a multivariate logistic regression to assess the association between racial and socioeconomic factors and the risk of PPD. Compared with White women, the adjusted odds ratio (OR) of PPD was significantly lower for Black women (OR 0.31, 95% CI 0.30-0.33), Asian or Pacific Islander women (OR 0.17, 95% CI 0.15-0.19), Hispanic women (OR 0.21, 95% CI 0.19-0.22), Native American women (OR 0.35, 95% CI 0.24-0.50), and women of other races and ethnicities (OR 0.38, 95% CI 0.34-0.42). Married women have significantly lower odds of PPD than women who were divorced (OR 1.99, 95% CI 1.71-2.31), legally separated (OR 1.97, 95% CI 1.60-2.41), single (OR 1.45, 95% CI 1.38-1.51), or widowed (OR 2.96, 95% CI 1.82-4.64). Women living in areas with a median household income <US $46,000 have higher odds of PPD than women living in areas with median household income >US $59,000 (OR 0.79, 95% CI 0.73-0.85). Compared with women who enrolled in private insurance, the odds of PPD were higher for women with Medicare (OR 3.40, 95% CI 2.69-4.26) and lower for women who self-paid (OR 0.70, 95% CI 0.58-0.84) or had no charge such as charities (OR 0.60, 95% CI 0.45-0.80). Compared with women living in metropolitan areas with population sizes >1 million, the odds of PPD were lower for those living in metropolitan areas with population sizes <250,000 (OR 0.60, 95% CI 0.54-0.67), lower for those living in urban areas with population sizes of 2500 to 19,999 (adjacent to a metropolitan area; OR 0.75, 95% CI 0.66-0.84), and higher for those living in metropolitan areas with population sizes of 250,000 to 1 million (OR 1.21, 95% CI 1.13-1.30). Women with higher Charlson Comorbidity Index scores had higher odds of PPD than those with lower Charlson Comorbidity Index scores (OR 1.47, 95% CI 1.43-1.52).

Table 3 presents the results of the multivariate Cox regression analysis. Compared with White women, the adjusted hazards of PPD were significantly lower for Black women (hazard ratio [HR] 0.34, 95% CI 0.33-0.36), Asian and Pacific Islander women (HR 0.21, 95% CI 0.19-0.24), Hispanic women (HR 0.27, 95% CI 0.25-0.29), Native American women (HR 0.36, 95% CI 0.25-0.52), and women of other races and ethnicities (HR 0.43, 95% CI 0.38-0.48). Women >35 years (HR 1.10, 95% CI 1.05-1.15) had higher hazards of PPD compared with those aged 20 to 35 years. Compared with married women, the hazards of PPD were significantly higher for women who were divorced (HR 1.78, 95% CI 1.55-2.05), legally separated (HR 1.75, 95% CI 1.44-2.12), single (HR 1.42, 95% CI 1.35-1.48), widowed (HR 2.84, 95% CI 1.87-4.32), or those with missing marital status records (HR 1.29, 95% CI 1.12-1.50). Compared with women who lived in areas with median household incomes of <US $46,000, the hazards were lower for women who lived in areas with median household incomes of US $59,000-78,999 (HR 0.85, 95% CI 0.80-0.92) and areas of income >US $79,000 (HR 0.86, 95% CI 0.81-0.93). Compared with women enrolled in private insurance, the hazards of PPD were higher for women enrolled in Medicare (HR 2.13, 95% CI 1.76-2.59) but lower for women who had no charges such as charities (HR 0.44, 95% CI 0.33-0.59) or self-paid (HR 0.56, 95% CI 0.47-0.67). Compared with women living in metropolitan areas with population sizes of >1 million, women living in metropolitan areas with population sizes of 250,000 to 1 million had higher hazards of PPD (HR 1.14, 95% CI 1.07-1.23). In contrast, the hazards of PPD were lower for those living in metropolitan areas with population size <250,000 (HR 0.57, 95% CI 0.51-0.64) and those living in urban areas with a population size of 2500 to 19,999 (adjacent to a metropolitan area; HR 0.73, 95% CI 0.65-0.82). Women with higher Charlson Comorbidity Index scores had higher hazards of PPD compared with those with lower Charlson Comorbidity Index scores (HR 1.19, 95% CI 1.17-1.23). The log-rank test suggested a significant difference in the timing of diagnosis among patients of different races and ethnicities at the 0.1% level (Table 4).

The results of the multinomial logistic regression are presented in Table 5. Compared with White women, the risk of diagnosis after 8 weeks relative to within 4 weeks was significantly higher for Black women (relative risk [RR] 2.12, 95% CI 1.73-2.60) and Asian and Pacific Islander women (RR 2.48, 95% CI 1.46-4.21). Compared with women enrolled in private insurance, women who had Medicaid (RR 1.69, 95% CI 1.37-2.10) or self-paid (RR 3.65, 95% CI 2.05-6.50) had higher risks of diagnosis after 8 weeks relative to before 4 weeks. Women who had no charge (such as charities or donations) had a higher risk of diagnosis after 4 to 8 weeks relative to before 4 weeks (RR 9.76, 95% CI 3.79-25.08). Compared with women living in metropolitan areas with population sizes >1 million, the risks of PPD in 4 to 8 weeks relative to before 4 weeks were lower for those living in metropolitan areas with population sizes of 250,000 to 1 million (RR 0.20, 95% CI 0.08-0.51). Compared with women living in metropolitan areas with population sizes of >1 million, the risks of PPD after 8 weeks relative to before 4 weeks were higher for those living adjacent to metropolitan areas with population sizes of 2500 to 19,999 (RR 2.15, 95% CI 1.42-3.25). Women who had higher Charlson Comorbidity Index scores exhibited higher risks of diagnosis in both 4 to 8 weeks (RR 1.34, 95% CI 1.14-1.56) and after 8 weeks (RR 1.28, 95% CI 1.16-1.41) relative to before 4 weeks (RR 1.34, 95% CI 1.14-1.56; RR 1.28, 95% CI 1.16-1.41).

**Table 2:** Multivariate logistic regression depicting the association between racial and socioeconomic factors and risk of postpartum depression.

Variable			Odds ratio (95% CI)		*P* value
**Age group (years)**					
	20-35	Ref^a^		N/A^b^	
	<20	1.05 (0.95-1.16)		.31	
	≥35	1.05 (1.01-1.10)		.02	
**Race**					
	White	Ref		N/A^b^	
	Black	0.31 (0.30-0.33)		<.001^c^	
	Hispanic	0.21 (0.19-0.22)		<.001^c^	
	Asian or Pacific Islander	0.17 (0.15-0.19)		<.001^c^	
	Native American	0.35 (0.24-0.50)		<.001^c^	
	Other	0.38 (0.34-0.42)		<.001^c^	
**Marital status**					
	Married	Ref		N/A^b^	
	Single	1.45 (1.38-1.51)		<.001^c^	
	Legally separated	1.97 (1.60-2.41)		<.001^c^	
	Divorced	1.99 (1.71-2.31)		<.001^c^	
	Widowed	2.96 (1.82-4.64)		<.001^c^	
	Other	1.26 (1.08-1.46)		.003	
**Zip-level median household income (US $)**					
	1-45,999	Ref		N/A^b^	
	46,000-58,999	0.98 (0.90-1.06)		.56	
	59,000-78,999	0.79 (0.73-0.85)		<.001^c^	
	>79,000	0.79 (0.74-0.85)		<.001^c^	
**Insurance type**					
	Private insurance	Ref		N/A^b^	
	Medicaid	1.08 (1.03-1.13)		.002	
	Medicare	3.40 (2.69-4.26)		<.001^c^	
	Self-pay	0.70 (0.58-0.84)		<.001^c^	
	No charge	0.60 (0.45-0.80)		<.001^c^	
	Other	1.16 (1.04-1.28)		.007	
**Urban or rural**					
	Metropolitan areas of ≥1 million population	Ref		N/A^b^	
	Metropolitan areas of 250,000 to 1 million population	1.21 (1.13-1.30)		<.001^c^	
	Metropolitan areas of <250,000 population	0.60 (0.54-0.67)		<.001^c^	
	Urban population of 2500 to 19,999, adjacent to a metropolitan area	0.75 (0.66-0.84)		<.001^c^	
	Urban population of 2500 to 19,999, not adjacent to a metropolitan area^d^	N/A		N/A	
	Completely rural or <2500 urban population, adjacent to a metropolitan area^d^	N/A		N/A	
Charlson Comorbidity Index score			1.47 (1.43-1.52)		<.001^c^

^a^Ref: reference group.

^b^N/A: not applicable.

^c^*P*<.001 indicates statistical significance.

^d^Estimates could not be calculated owing to small sample size.

**Table 3:** Multivariate Cox regression depicting the association between the racial and socioeconomic factors and hazards of postpartum depression.

Variable			Hazard ratio (95% CI)		*P* value
**Age group (years)**					
	20-35	Ref^a^		N/A^b^	
	<20	1.11 (1.01-1.23)		.03	
	≥35	1.10 (1.05-1.15)		<.001^c^	
**Race**					
	White	Ref		N/A^b^	
	Black	0.34 (0.33-0.36)		<.001^c^	
	Hispanic	0.27 (0.25-0.29)		<.001^c^	
	Asian or Pacific Islander	0.21 (0.19-0.24)		<.001^c^	
	Native American	0.36 (0.25-0.52)		<.001^c^	
	Other	0.43 (0.38-0.48)		<.001^c^	
**Marital status**					
	Married	Ref		N/A^b^	
	Single	1.42 (1.35-1.48)		<.001^c^	
	Legally separated	1.75 (1.44-2.12)		<.001^c^	
	Divorced	1.78 (1.55-2.05)		<.001^c^	
	Widowed	2.84 (1.87-4.32)		<.001^c^	
	Other	1.29 (1.12-1.50)		<.001^c^	
**Zip-level median household income (US $)**					
	1-45,999	Ref		N/A^b^	
	46,000-58,999	1.02 (0.94-1.11)		.58	
	59,000-78,999	0.85 (0.80-0.92)		<.001^c^	
	>79,000	0.86 (0.81-0.93)		<.001^c^	
**Insurance type**					
	Private insurance	Ref		N/A^b^	
	Medicaid	0.96 (0.92-1.01)		.11	
	Medicare	2.13 (1.76-2.59)		<.001^c^	
	Self-pay	0.56 (0.47-0.67)		<.001^c^	
	No charge	0.44 (0.33-0.59)		<.001^c^	
	Other	1.11 (1.00-1.23)		.06	
**Urban or rural**					
	Metropolitan areas of ≥1 million population	Ref		N/A^b^	
	Metropolitan areas of 250,000 to 1 million population	1.14 (1.07-1.23)		<.001^c^	
	Metropolitan areas of <250,000 population	0.57 (0.51-0.64)		<.001^c^	
	Urban population of 2500 to 19,999, adjacent to a metropolitan area	0.73 (0.65-0.82)		<.001^c^	
	Urban population of 2500 to 19,999, not adjacent to a metropolitan area^d^	N/A		N/A	
	Completely rural or <2500 urban population, adjacent to a metropolitan area^d^	N/A		N/A	
Charlson Comorbidity Index score			1.19 (1.17-1.23)		<.001^c^

^a^Ref: reference group.

^b^N/A: not applicable.

^c^*P*<.001 indicates statistical significance.

^d^Estimates could not be calculated owing to small sample size.

**Table 4:** Log-rank test depicting the survival by race groups.

Log-rank test	Chi-square *(df)*	*P* value
White versus Black	1676 (1)	<.001^a^
White versus Asian or Pacific Islander	785 (1)	<.001^a^
White versus Hispanic	1676 (1)	<.001^a^
White versus Native American	36.2 (1)	<.001^a^
White versus other	241 (1)	<.001^a^

^a^*P*<.001 indicates statistical significance.

**Table 5:** Multivariate multinomial regression depicting the association between the racial and socioeconomic factors and the timing of postpartum depression diagnosis (between 4 and 8 weeks and after 8 weeks compared with before 4 weeks).

Variable		After 8 weeks, RR^a^ (95% CI)	*P* value	Between 4 and 8 weeks, RR (95% CI)	*P* value
**Age group** **(years)**					
	20-35	Ref^b^	N/A^c^	N/A^c^	N/A^c^
	<20	0.91 (0.63-1.32)	.62	0.84 (0.42-1.67)	.61
	≥35	0.98 (0.78-1.22)	.82	1.22 (0.85-1.74)	.28
**Race**					
	White	Ref	N/A^c^	N/A^c^	N/A^c^
	Black	2.12 (1.73-2.60)	<.001^d^	1.55 (1.07-2.23)	.02
	Hispanic	1.32 (0.9-1.83)	.10	1.31 (0.75-2.28)	.34
	Asian or Pacific Islander	2.48 (1.46-4.21)	<.001^d^	3.19 (1.44-7.04)	.004
	Native American	1.06 (0.14-7.87)	.96	3.05 (0.40-23.07)	.28
	Other	1.19 (0.71-2.00)	.51	1.96 (0.97-3.96)	.06
**Marital status**					
	Married	Ref	N/A^c^	N/A^c^	N/A^c^
	Single	1.34 (1.08-1.66)	.008	1.47 (1.02-2.12)	.04
	Legally separated	1.76 (0.89-3.50)	.11	0.64 (0.09-4.73)	.66
	Divorced	1.24 (0.66-2.34)	.51	0.70 (0.17-2.93)	.63
	Widowed^e^	0.99 (0.13-7.41)	.99	N/A^e^	N/A^e^
	Other	0.71 (0.31-1.64)	.42	2.78 (1.32-5.90)	.007
**Zip-level median household income** **(US $)**					
	1-45,999	Ref	N/A^c^	N/A^c^	N/A^c^
	46,000-58,999	1.00 (0.73-1.37)	.98	2.15 (1.20-3.86)	.01
	59,000-78,999	1.02 (0.77-1.34)	.91	1.55 (0.91-2.65)	.11
	>79,000	1.00 (0.75-1.33)	.99	0.97 (0.56-1.68)	.9
**Insurance type**					
	Private insurance	Ref	N/A^c^	N/A^c^	N/A^c^
	Medicaid	1.69 (1.37-2.10)	<.001^d^	1.32 (0.92-1.90)	.13
	Medicare	1.87 (0.94-3.70)	.07	N/A^e^	N/A^e^
	Self-pay	3.65 (2.05-6.50)	<.001^d^	2.29 (0.80-6.57)	.12
	No charge	1.51 (0.35-6.57)	.58	9.76 (3.79-25.08)	<.001^d^
	Other	2.00 (1.29-3.10)	.002	0.62 (0.19-1.97)	.41
**Urban or rural**					
	Metropolitan areas of ≥1 million population	Ref	N/A^c^	N/A^c^	N/A^c^
	Metropolitan areas of 250,000 to 1 million population	1.20 (0.89-1.62)	.22	0.20 (0.08-0.51)	<.001^d^
	Metropolitan areas of <250,000 population	1.32 (0.83-2.10)	.24	2.23 (1.17-4.24)	.01
	Urban population of 2500 to 19,999, adjacent to a metropolitan area	2.15 (1.42-3.25)	<.001^d^	0.45 (0.14-1.44)	.18
	Urban population of 2500 to 19,999, not adjacent to a metropolitan area^e^	N/A	N/A	N/A	N/A
	Completely rural or <2500 urban population, adjacent to a metropolitan area^e^	N/A	N/A	N/A	N/A
Charlson Comorbidity Index score		1.28 (1.16-1.41)	<.001^d^	1.34 (1.14-1.56)	<.001^d^

^a^RR: relative risk.

^b^Ref: reference group.

^c^N/A: not applicable.

^d^*P*<.001 indicates statistical significance.

^e^Estimates could not be calculated owing to small sample size.

### Sensitivity Analyses

The results of the post hoc sensitivity analyses are presented in Multimedia Appendix 2. Additional regression models that included patients with prior depression and those that excluded patients who had depression diagnosed during the same stay as childbirth produced estimates close to our main analysis. Hence, the direction of our findings remains consistent.

## Discussion

### Principal Findings

This study investigated the disparate timing of PPD among individuals who had an inpatient delivery in Maryland from 2017 to 2019. We performed logistic regression to evaluate the adjusted odds of PPD among the races and ethnicities. In addition, we performed multinomial and Cox regression analyses to examine the timing of PPD, adjusted for demographics, socioeconomic characteristics, and comorbidities. Disparate timing of PPD diagnosis could inform strategies to identify the most vulnerable patients and initiate treatment promptly.

The odds of PPD are significantly higher among individuals who are White, with more comorbid conditions, without a partner, with Medicare, with residential addresses in areas of lower median household income, and from metropolitan areas of population sizes 250,000 to 1 million. We found lower odds of PPD for individuals who were Black or Hispanic, contradicting previous literature that found higher odds [11,25]. Differences in findings could be attributed to four factors: (1) demographic composition, (2) data sources and quality, (3) varying health care access, and (4) culture. Maryland has the fifth largest Black or African American population (nearly 30%) [26]; thus, the population could be meaningfully different to start with compared with previous studies, which used data from other states such as California [9]. In addition to inherent differences between data sources, the disparity in health care access among racial groups may also affect data quality and completeness. In other words, sources of disparity include both the underlying racial and socioeconomic population as well as data quality. Individuals without proper access to hospital and PPD care would not be recorded in the HCUP databases in the first place. Therefore, the HCUP database only captures individuals who can access a hospital facility, thus possibly skewing the results across socioeconomic strata. Finally, sociocultural barriers such as stigma, lack of social support, and adversities to mothering could also explain the lower odds among Black and Hispanic individuals [27,28]. Fears of negative perception and stigma could impede individuals from reporting symptoms to their care provider, and a lack of social support and adversities to mothering (poverty, marital status, and income) create additional burdens for receiving appropriate care.

Cox regression also found significantly higher hazards for individuals who were White, with more comorbid conditions, without a partner, with Medicare, with residential addresses in areas with lower median household income, and from metropolitan areas of population sizes 250,000 to 1 million. Similarly, results from the multinomial regression suggest that, among individuals with PPD, the odds of delayed diagnoses are significantly higher for those who are not White, with residential addresses in areas with higher median household income, and those enrolled in public insurance.

The findings from both analyses indicate significant associations between being single and having higher risk of PPD. Having a partner typically means additional mental and financial support during and after childbirth. In contrast, giving birth to a child without additional support could be a huge burden. As shown in Table S1 in Multimedia Appendix 2, races exhibited different patterns of marital status. This could be interpreted as the manifestation of cultural differences regarding pregnancy and marriage. Going through pregnancy and delivery without additional support increases the burden of childbirth and the risk of PPD [28,29].

From multinomial regression, we found disparate timing of PPD diagnosis, which could be explained by cultural perceptions of PPD. Previous studies have shown hesitancy to seek PPD treatment as a common theme [3,4]. Furthermore, multiple studies have shown hesitancy to seek treatment could have manifested through social, behavioral, and financial barriers (ie, fear of judgment, social support, financial cost, and transportation) [15,28]. These same burdens could also explain why racialized individuals present later in the postpartum period. Racialized individuals were likely to cope with said social, behavioral, and financial challenges before receiving care, if any. As a result, there could be a gap between when individuals conceive of help-seeking thoughts or behaviors and when they receive the care, hence delaying effective treatment. A higher median household income at the zip-level might reflect better access to care rather than delayed diagnoses. Similarly, urban or rural areas themselves are not direct risk factors for delayed diagnoses, but they reflect the extent of patient capture or coverage. On the one hand, patients may present later to the health care system, but they are captured at the least. On the other hand, patients with greater health care access barriers may not be captured at all. Finally, in contrast to the previous literature, we found inconclusive evidence that individuals with Medicaid were diagnosed earlier than those with Medicare [14]. However, this could be attributed to the small sample size, as we only had 410 patients with Medicare (ie, patients with disability, end-stage renal disease, or amyotrophic lateral sclerosis) who delivered in the study time frame. Taken together, the log-rank test, Cox regression, and multinomial regression concur that the risks of PPD and timing of diagnosis vary by race group, and non-White race groups experience higher risks of delayed diagnosis. In turn, delayed treatment increases the chances of poor health outcomes.

Disparate timing in diagnosing PPD calls for alternative phenotyping strategies to provide higher and earlier screening rates for individuals with certain racial, sociodemographic, and economic backgrounds. Previous studies did not find the best screening tool or the best time duration for screening [30]. A recent study suggested that early screening for PPD should be coupled with multiple follow-ups in the year after delivery [31]. Our findings suggest that racial groups such as Black, Asian, and Hispanic should be screened earlier as they are more likely to have delayed PPD diagnoses. Instead of screening all individuals at the same rate, a targeted approach would conserve resources for the vulnerable populations with the highest risks.

This study has several policy implications. Various state-level policies support PPD screening and interventions. According to a 2015 study, 13 states have enacted mandates to address education, screening, awareness, and state-level reporting in patients with PPD [32]. One of the most promising intervention strategies is offering home visits [32]. What could enhance this approach is accounting for the racial disparity in PPD diagnosis as well as timing. If state policies could mandate insurance coverage for PPD home visits, this could overcome some of the hesitancy to seek treatment and provide timely care.

### Limitations

Our study had a few limitations. First, our data source is based in Maryland, which has a higher Black population than the national average. Consequently, our findings may not be generalizable to states with different demographic characteristics. Second, our study is limited by the quality of data captured in the HCUP data sets. HCUP reports races and ethnicities together; thus, no ethnic distinctions are made for the race groups in our study. As previously mentioned, a high proportion of PPD cases is unreported or undiagnosed. Even in the absence of a hospital PPD diagnosis, individuals could have developed PPD without a subsequent hospital visit. Moreover, our study did not include the PPD records in outpatient settings such as mental health facilities or obstetrics and gynecology clinics. Therefore, the overall PPD rates across the population or different racial groups could have been underreported in this study. Third, we applied the Cox regression model to measure the risk differences between racial groups during the postpartum period, but other methods, such as the Cure model, might be more flexible for the assumptions made in this study [33]. Fourth, hierarchical models are typically used to model group-level effects such as median household income (ie, zip-level); however, our multilevel analysis resulted in findings that resemble those from the multivariate logistic regression. Finally, this study did not distinguish between PPD after a cesarean section and a vaginal delivery, although studies have shown that cesarean sections significantly increased the risk of PPD [34]. Future studies analyzing the disparate risks of PPD should differentiate between the types of delivery.

### Future Work

There are 3 considerations for future studies to build upon the findings of this study. First, to the best of our knowledge, few existing studies have acknowledged the impact of data quality on the results. As discussed, not only could there be racial and socioeconomic disparities, but disparities could also arise from data quality. To guide clinical practice and intervention policies, the impact of data quality must be further evaluated. Second, additional work is needed on sociobehavioral factors related to PPD as this is not well understood at present. This could support targeted strategies for diagnosis and treatment initiations. Finally, future research should consider a combination of data sources when studying PPD. The Pregnancy Risk Assessment Monitoring System is the only database that tracks the occurrence of screening across states (81% of all deliveries) [8]. As there is a lack of a central database for all delivery-related data, future studies should consider using multiple data sources to analyze racial disparities in PPD diagnoses.

### Conclusions

This study aimed to address the underlying racial disparities in PPD phenotyping and to provide targeted and timely care. We found significant racial disparities in the risk and timing of PPD diagnosis. Compared with White individuals, Black, Hispanic, and Asian individuals have lower odds of PPD, and Black and Asian individuals are more likely to have a PPD diagnosis later in the postpartum period. Diagnosis of patients with potential PPD should account for the disparate risks and timing among races and ethnicities. Our findings serve to enhance intervention strategies and health care policies as well as highlight data and informatics needs to support future work on racial disparities in PPD.

## Appendix

Multimedia Appendix 1 Postpartum Depression ICD-10 Codes.

Multimedia Appendix 2 Sensitivity Analysis Results.

## Abbreviations

HCUP: Healthcare Cost and Utilization Project
HR: hazard ratio
ICD-10: International Classification of Diseases
OR: odds ratio
PPD: postpartum depression
RR: relative risk
