# Correction: Assessing the Racial and Socioeconomic Disparities in Postpartum Depression Using Population-Level Hospital Discharge Data: Longitudinal Retrospective Study

**DOI:** 10.2196/66676

**Published:** 2024-10-09

**Authors:** Star Liu, Xiyu Ding, Anas Belouali, Haibin Bai, Kanimozhi Raja, Hadi Kharrazi

**Affiliations:** 1 Johns Hopkins University School of Medicine Baltimore, MD United States; 2 Johns Hopkins University Bloomberg School of Public Health Baltimore, MD United States

In “Assessing the Racial and Socioeconomic Disparities in Postpartum Depression Using Population-Level Hospital Discharge Data: Longitudinal Retrospective Study” JMIR Pediatr Parent 2022;5(4):e38879 the authors noted 4 errors.

In “Results”, first paragraph, the numbers in parentheses “(66,939/160,066)” have been revised to “(65,028/160,066)”, as follows:

Of the study population, 40.63% (65,028/160,066) were White,...

In “Results”, first paragraph, the numbers in parentheses “(26,360/160,066, 16.47%)” have been revised to “(12,658/48,953, 25.86%)”, as follows:

Among all racial groups, the Black population had the highest proportion of individuals living in areas with <US $59,000 median household income (12,658/48,953, 25.86%).

Under Results, third paragraph, the word “higher” has been revised to “lower”, as follows:

Married women have significantly lower odds of PPD than women who were divorced (OR 1.99, 95% CI 1.71-2.31), legally separated (OR 1.97, 95% CI 1.60-2.41), single (OR 1.45, 95% CI 1.38-1.51), or widowed (OR 2.96, 95% CI 1.82-4.64).

Under Results, third paragraph, the word “lower” has been replaced by “higher” in the sentence:

Women living in areas with a median household income <US $46,000 have higher odds of PPD than women living in areas with median household income >US $59,000 (OR 0.79, 95% CI 0.73-0.85).

The correction will appear in the online version of the paper on the JMIR Publications website on October 9, 2024, together with the publication of this correction notice. Because this was made after submission to PubMed, PubMed Central, and other full-text repositories, the corrected article has also been resubmitted to those repositories.

