# Supplementary material for: Assessing the Racial and Socioeconomic Disparities in Postpartum Depression Using Population-Level Hospital Discharge Data: Longitudinal Retrospective Study
Source: JMIR Pediatr Parent. 2022 Oct 17;5(4):e38879. doi: 10.2196/38879 (PMC9623466; doi:10.2196/38879)
Supplement: Multimedia Appendix 1 [file pediatrics_v5i4e38879_app1.docx]

**Multimedia Appendix 1:** Postpartum Depression ICD-10 Codes.

| **Postpartum Depression ICD-10 Codes** |
| --- |
| F53 Puerperal psychosis |
| O906 Postpartum mood disturbance |
| O99340 Other mental disorders complicating pregnancy, unspecified trimester |
| O99341 Other mental disorders complicating pregnancy, first trimester |
| O99342 Other mental disorders complicating pregnancy, second trimester |
| O99343 Other mental disorders complicating pregnancy, third trimester |
| O99344 Other mental disorders complicating childbirth |
| O99345 Other mental disorders complicating the puerperium |
| F99 Mental disorder, not otherwise specified |
| F320 Major depressive disorder, single episode, mild |
| F321 Major depressive disorder, single episode, moderate |
| F322 Major depressive disorder, single episode, severe w/o psychotic features |
| F323 Major depressive disorder, single episode, severe w psychotic features |
| F324 Major depressive disorder, single episode, in partial remission |
| F325 Major depressive disorder, single episode, in full remission |
| F328 Other depressive episodes |
| F329 Major depressive disorder, single episode, unspecified |
| F330 Major depressive disorder, recurrent, mild |
| F331 Major depressive disorder, recurrent, moderate |
| F332 Major depressive disorder, recurrent severe without psychotic features |
| F333 Major depressive disorder, recurrent, severe with psychotic symptoms |
| F3340 Major depressive disorder, recurrent, in remission, unspecified |
| F3341 Major depressive disorder, recurrent, in partial remission |
| F3342 Major depressive disorder, recurrent, in full remission |
| F338 Other recurrent depressive disorders |
| F339 Major depressive disorder, recurrent, unspecified |
