# Supplementary material for: Assessing the Racial and Socioeconomic Disparities in Postpartum Depression Using Population-Level Hospital Discharge Data: Longitudinal Retrospective Study
Source: JMIR Pediatr Parent. 2022 Oct 17;5(4):e38879. doi: 10.2196/38879 (PMC9623466; doi:10.2196/38879)
Supplement: Multimedia Appendix 2 [file pediatrics_v5i4e38879_app2.docx]

**Multimedia Appendix 2:** Sensitivity Analysis Results.

Appendix 2 Table 1. Population demographics are stratified by race groups.


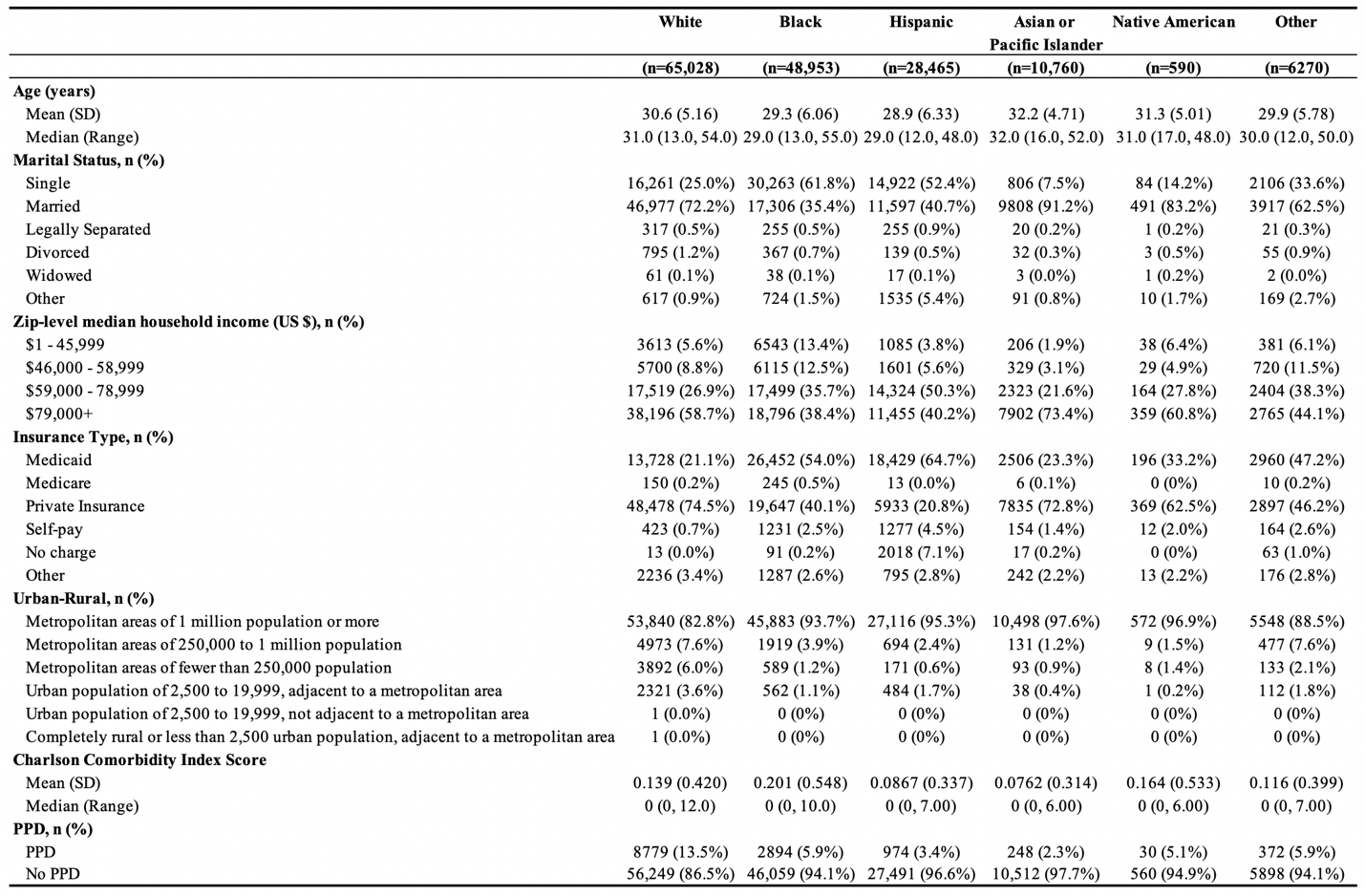


As shown in Appendix 2 Table 1, noticeable differences exist among the race groups. The Asian population is older (mean age 32.2). The Black and Hispanic populations have the lowest proportion of married individuals (35.4% and 40.7%, respectively). Those populations also have lower proportions of individuals residing in areas with the highest median household income ($79,000+). As a result, the Black and Hispanic populations have higher proportions of individuals enrolled in Medicaid rather than paying for private insurance. This highlights both cultural and socioeconomic differences among the race groups. These differences could contribute to the disparity in the risk and timing of PPD.

Appendix 2 Table 2. Multivariate logistic regression including individuals with a depression diagnosis within the prior year.


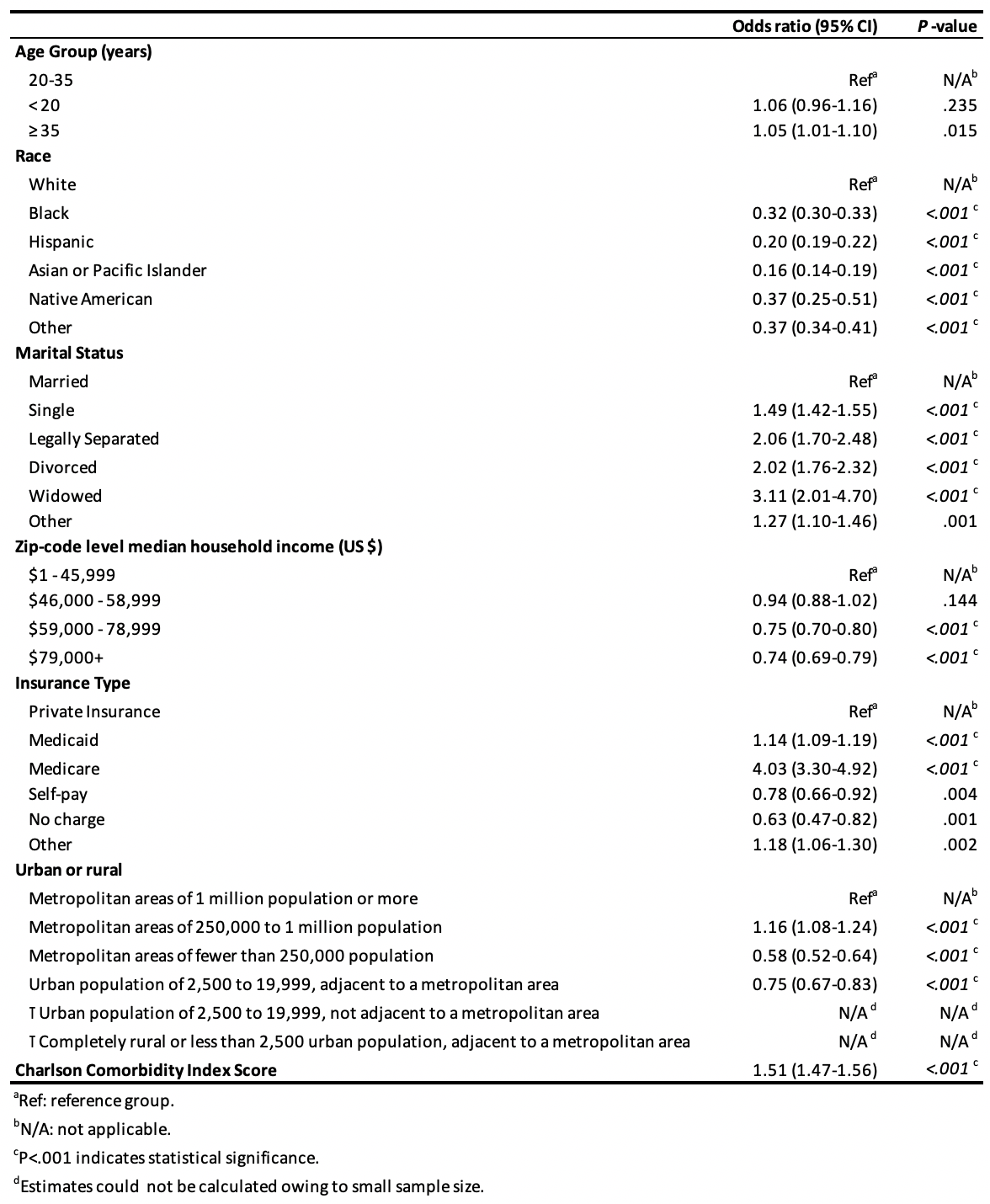


As shown in Appendix 2 Table 2, the multivariate logistic regression model, which includes individuals with a depression diagnosis in the prior year, has estimates that are close to the multivariate logistic regression model that excluded those individuals.

Appendix 2 Table 3. Multivariate logistic regression including individuals with a depression diagnosis within the prior year and excluding PPD diagnosis that occurred on the day of delivery.


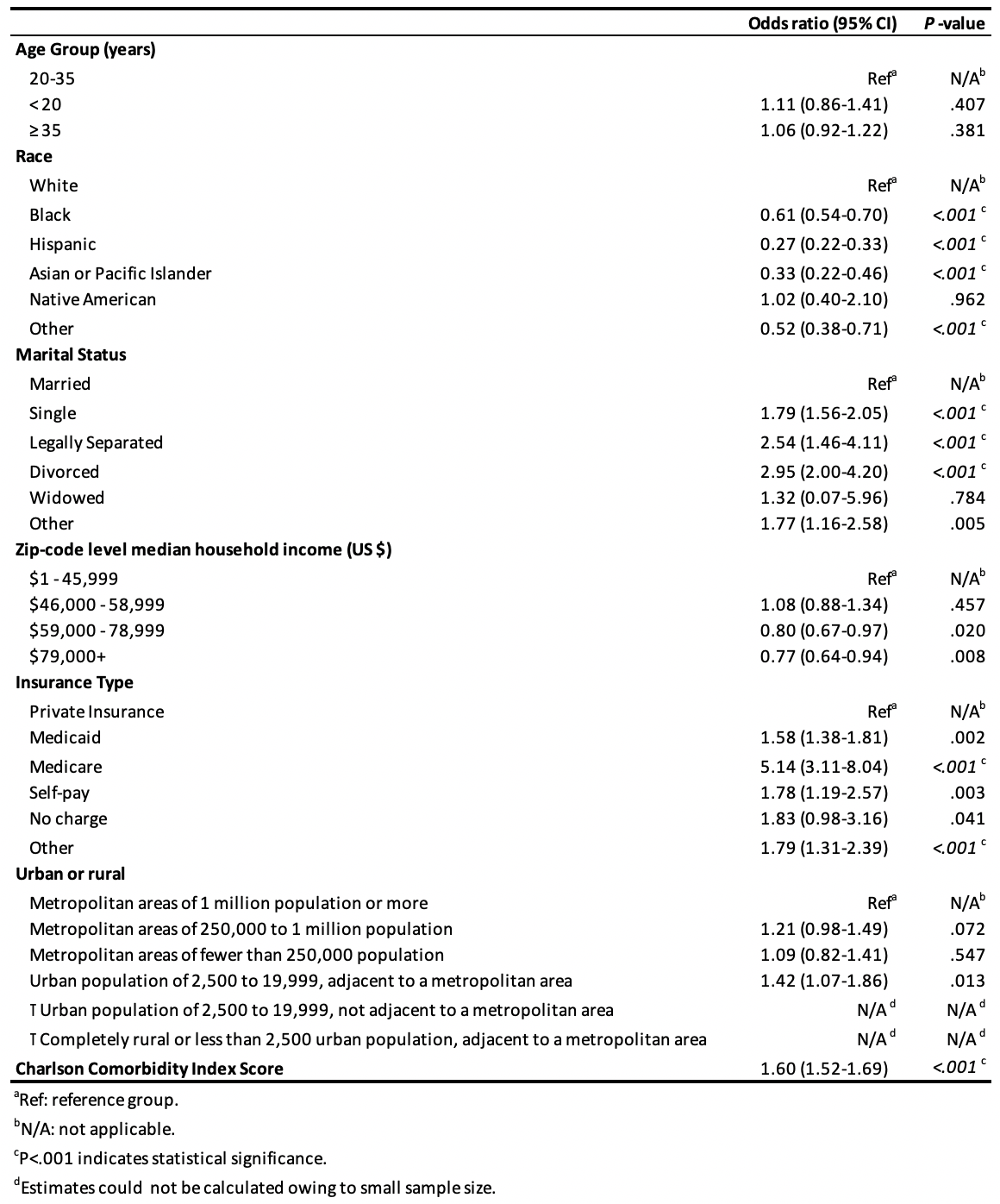


As shown in Appendix 2 Table 3, this multivariate logistic regression model differs from the model in Table B2 by excluding PPD diagnosis that occurred on the day of delivery. This model’s estimates are close to the multivariate logistic regression model that included PPD diagnosis that occurred on day one.

Appendix 2 Table 4. Multivariate Cox regression including individuals with a depression diagnosis within the prior year.


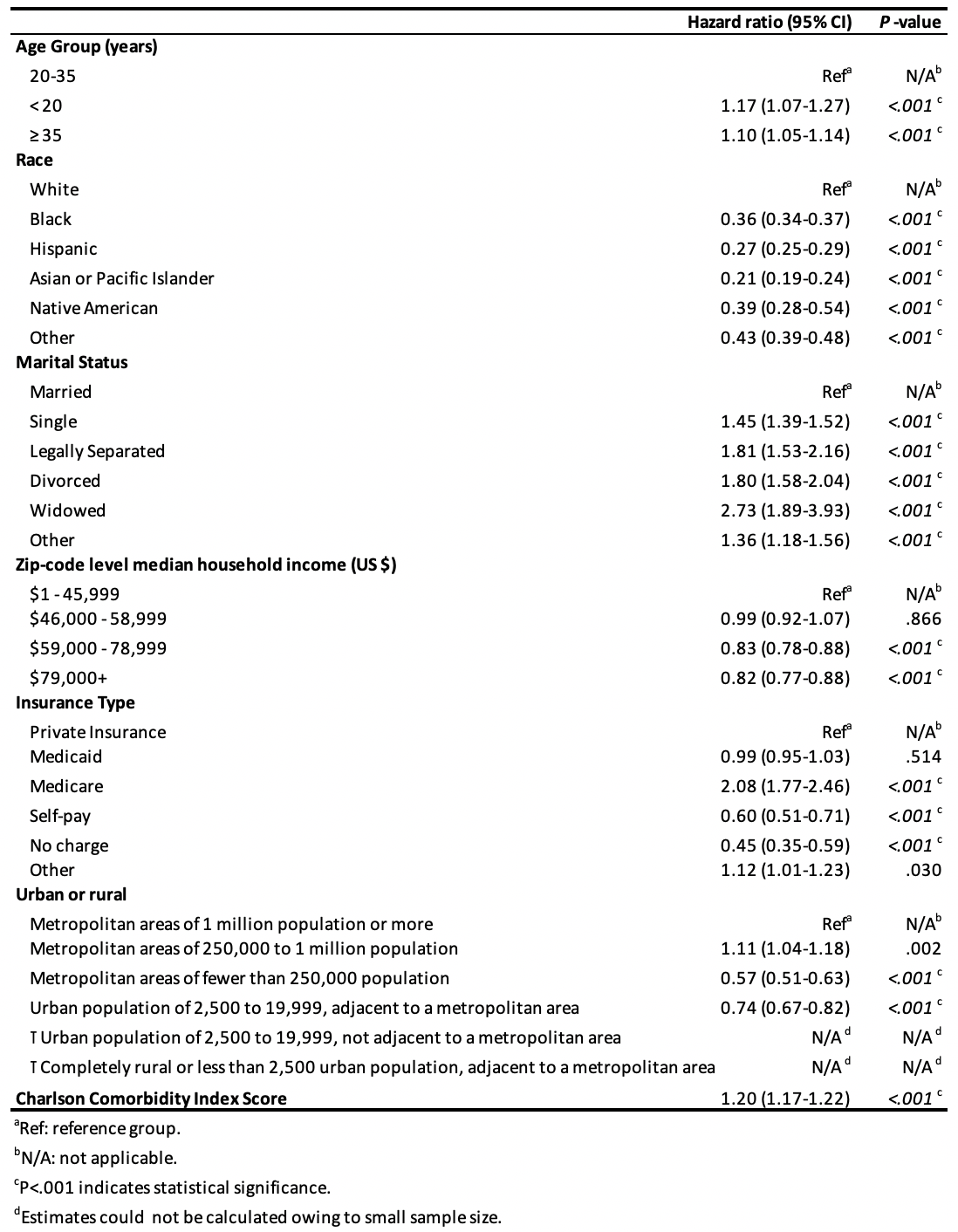


As shown in Appendix 2 Table 4, this multivariate Cox regression model, which includes individuals with a depression diagnosis in the prior year, has estimates that are close to the Cox regression model that excluded individuals with prior depression.

Appendix 2 Table 5. Multivariate multinomial regression including individuals with a depression diagnosis within the prior year.


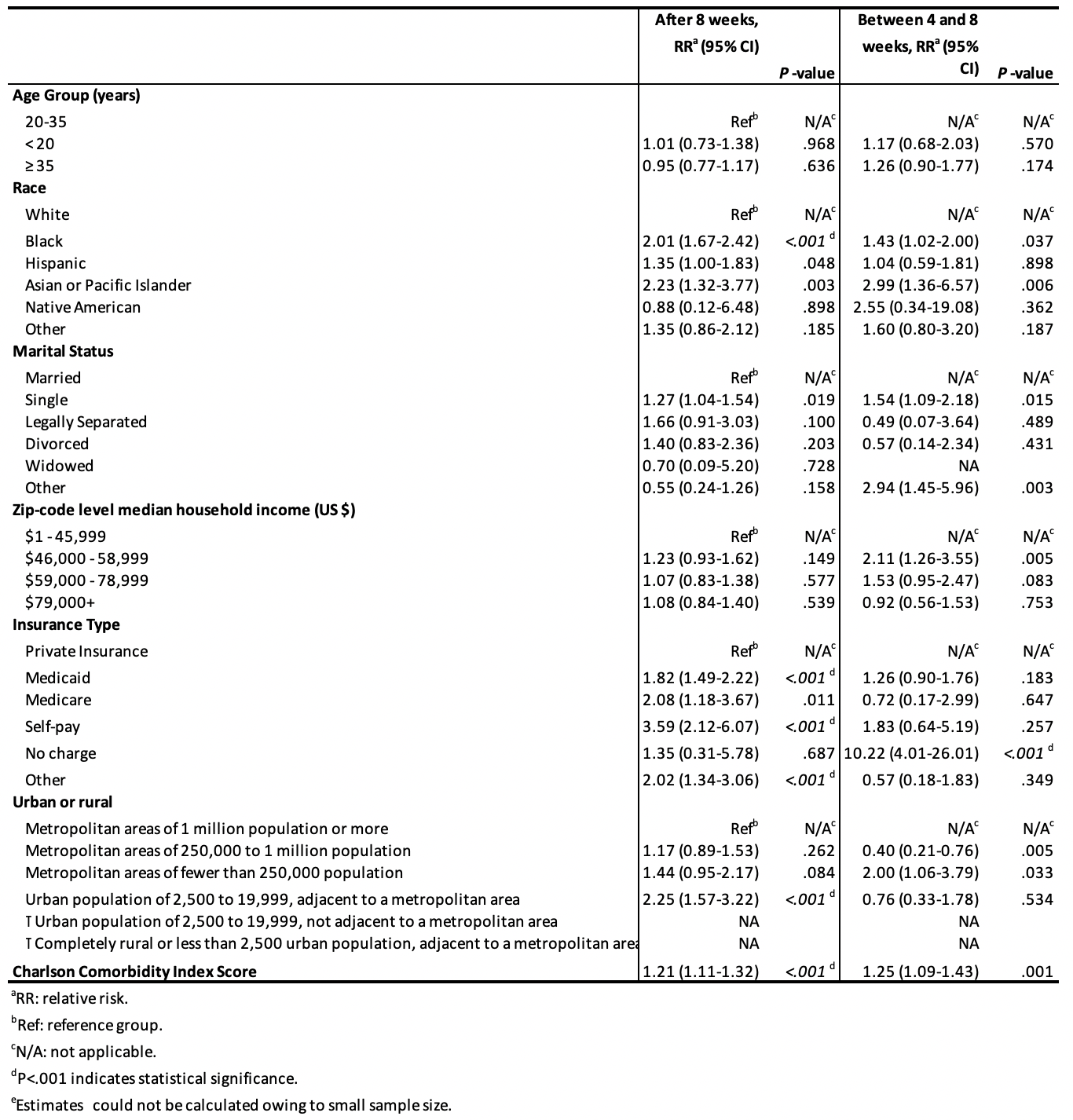


As shown in Appendix 2 Table 5, this multivariate multinomial regression model, which includes individuals with a depression diagnosis in the prior year, has estimates that are close to the multivariate multinomial regression model that excluded individuals with prior depression.
